# Prevention and management of musculoskeletal pain in nursing staff by a multifaceted intervention in the workplace: design of a cluster randomized controlled trial with effectiveness, process and economic evaluation (INTEVAL_Spain)

**DOI:** 10.1186/s12889-019-6683-7

**Published:** 2019-03-28

**Authors:** Consol Serra, Mercè Soler-Font, Ana María García, Pilar Peña, Sergio Vargas-Prada, José María Ramada

**Affiliations:** 1 Centre for Research in Occupational Health, Pompeu Fabra University/IMIM-Hospital del Mar Medical Research Institute, PRBB-Barcelona Biomedical Research Park. Dr. Aiguader, 88, 08003 Barcelona, Spain; 2 CIBER of Epidemiology and Public Health, Barcelona, Spain; 3 Occupational Health Service, Parc de Salut Mar, Barcelona, Spain; 4 Department of Public Health, University of Valencia, Valencia, Spain; 5 Occupational Health Service, Corporació Sanitària Parc Taulí, Sabadell, Spain; 6 Healthy Working Lives Group, Institute of Health and Wellbeing, College of Medical, Veterinary and Life Sciences, University of Glasgow, Glasgow, UK; 7 Salus Occupational Health, Safety and Return to Work Services, NHS Lanarkshire, Hamilton, UK

**Keywords:** Musculoskeletal pain, Return to work, Cluster randomized controlled trial, Sick leave, Multifaceted intervention, Ergonomics, Case management, Health promotion

## Abstract

**Background:**

Musculoskeletal pain (MSP) is the leading cause of years lived with disability. In consequence, to reduce MSP and its associated sickness absence is a major challenge. Previous interventions have been developed to reduce MSP and improve return to work of workers with MSP, but combined approaches and exhaustive evaluation are needed. The objective of the INTEVAL_Spain project is to evaluate the effectiveness of a multifaceted intervention in the workplace to prevent and manage MSP in nursing staff.

**Methods:**

The study is designed as a two-armed cluster randomized controlled trial with a late intervention control group. The hospital units are the clusters of randomization and participants are nurses and aides. An evidence-based multi-component intervention was designed combining participatory ergonomics, case management and health promotion. Both the intervention and the control groups receive occupational health care as usual. Data are collected at baseline, and after six and 12 months. The primary outcomes are prevalence of MSP and incidence and duration of sickness absence due to MSP. Secondary outcomes are work role functioning and organizational preventive culture. The intervention process will be assessed through quantitative indicators of recruitment, context, reach, dose supplied, dose received, fidelity and satisfaction, and qualitative approaches including discussion groups of participants and experts. The economic evaluation will include cost-effectiveness and cost-utility, calculated from the societal and the National Health System perspectives.

**Discussion:**

Workplace health programs are one of the best options for the prevention and control of non-communicable diseases. The main feature of this study is its multifaceted, multidisciplinary and de-medicalized intervention, which encompasses three evidence-based interventions and covers all three levels of prevention, which have not been previously unified in a single intervention. Also, it includes a comprehensive quantitative and qualitative evaluation of the intervention process, health results, and economic impact. This study could open the possibility of a new paradigm for the prevention and management of MSP and associated sickness absence approach at the workplace.

**Trial registration:**

Current Controlled Trials ISRCTN15780649 Retrospectively registered 13th July 2018.

## Background

Musculoskeletal pain (MSP) and associated limitations in mobility and functional capacity are essential characteristics of musculoskeletal disorders (MSDs) [1]. Upper extremity and low back are the most common pain locations. During the last two decades low back pain has been the leading cause of years lived with disability, and its global prevalence and incidence still show an increasing trend [2]. Healthcare workers are an occupational group at high risk of developing MSP [3–5].

In Europe, there is evidence that around 70–80% of workers report discomfort due to awkward postures and forceful work [2, 3] with an impact not only on health but also on work and economy, representing 50% of sickness absence episodes and 60% of permanent disabilities [6]. This scenario generates new challenges on health systems, requiring new strategies for its prevention and management [7, 8].

International variations of the distribution of MSP in the population support that its onset and persistence are influenced by a complex and dynamic interaction between biological, psychosocial, cultural and also individual factors [9–11]. The biopsychosocial model simultaneously considers all these factors and their impact on health and general well-being [12]. Although this model has become the dominant framework through which the etiology and prognosis of MSDs is conceptualized, its application into practice has not been optimal [13]. Traditionally, the causes of MSDs, and also MSP, have been investigated through biomechanics, physiology, genetics, epidemiology and rehabilitation, but separated from other involved relevant disciplines [14]. This fragmented approach does not offer optimal management. Evidence suggests that a combination of several specific approaches in multi-component interventions that address various determinants of the problem and those that incorporate a workers’ participatory approach in the intervention process can obtain better results [15–17].

The INTEVAL_Spain is an evidence-based intervention and consists of three components: participatory ergonomics (primary prevention of occupational risk factors), case management (secondary and tertiary prevention) and promotion of healthy lifestyles at work. There is an increasing concern on the so-called participatory ergonomics programs because they are both multi-factorial and participatory [18] and are defined as interventions in the workplace in which the relevant company agents actively participate with the aims to identify and act over the determinants of MSDs [19, 20]. Participatory ergonomics programs have been tested in Spanish companies with promising results [21, 22]. There is scientific evidence that the management of musculoskeletal symptoms through a tailored approach could be more efficient if individuals are stratified according to certain prognostic profiles in the initial stages of the disorder and after treatment [23, 24]. At tertiary prevention, the available scientific evidence suggests that case management can reduce the duration of sickness absence, musculoskeletal symptoms and disability, and improve work continuity [25–27]. Integrating activities on healthy lifestyles at work is also an important component of occupational health programs to reduce MSP [28], so different strategies promoting workers’ physical activity, emotional wellbeing and healthy diet [29–32] were also included. As for healthcare workers, promoting healthy lifestyles should be a priority because of its double impact on their own health and that of patients, encouraging these lifestyles within the general population. In addition, the World Health Organization considers workplace as one of the best contexts for the prevention and control of non-communicable diseases [33].

This paper presents the design of a study that evaluates the multifaceted INTEVAL_Spain intervention in the workplace in nursing staff from two tertiary hospitals to prevent and manage MSP and its associated sickness absence. We hypothesize that this intervention will be effective in terms of reducing the prevalence of MSP and will also reduce the incidence of associated sickness absence and the time to return to work in the intervention group at the end of the study, compared to the control non-intervention group. In addition, a process evaluation of the intervention and its association with the impact on MSP, and an economic evaluation will be carried out.

## Methods

The CONSORT statement and the extension for cluster randomized trials were used to describe the design of the study [34, 35].

### Study design and context

**Fig. 1:**
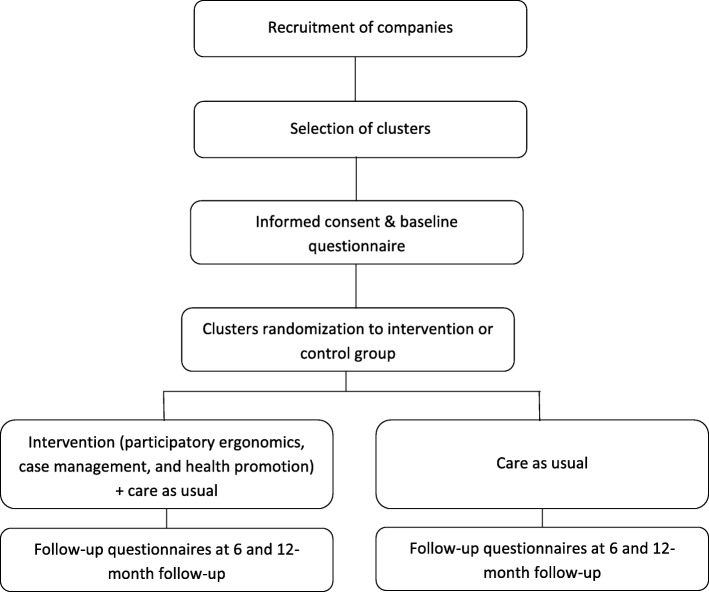
Flowchart and overview of the trial

The study is designed as a two-armed cluster randomized trial with a late intervention control group (Fig. 1) where clusters are independent hospital units, and participants are nursing staff (nurses and aides) highly exposed to ergonomic risk factors at work. There is extensive scientific evidence that incidence and prevalence of MSP is very high and is the leading cause of sickness absence in healthcare workers, especially nurses and aides working in hospitals [36], as it is exposure to ergonomic and other risk factors [37, 38]. It is also well known that other non-occupational, cultural and individual factors also play an important role in the incidence and prognosis of MSP [36]. In Spain, as in other European countries, all employers are required to organize some kind of occupational health service (OHS) according to exposure to occupational risks and company size. The tasks of these OHS include traditional risk assessment and investigation of occupational injuries, health surveillance mainly through health examinations, prevention, training and information covering occupational and non-occupational health risks. External OHS are usually the main occupational health provider for small and medium size companies, whereas large companies, including hospitals, usually have an in-house OHS which offer better opportunities for research and testing new approaches to improve workers‘health.

The study is conducted in two main tertiary hospitals in the cities of Barcelona and Sabadell (Barcelona province) with similar level of health care complexity, a workforce of around 4000 workers each, of whom around 60% are nursing staff, and with an experienced in-house OHS each, that share common methodologies and good coordination.

### Recruitment of companies

Hospitals had to meet the following criteria to guarantee the selection of appropriate clusters and the implementation of the intervention: (1) workforce of at least 500 workers, (2) commitment and explicit interest from the hospital management to carry out the intervention and its evaluation; (3) existence of an in-house OHS that maintains routine quality records and is interested in developing the intervention and; (4) existence of work units with exposure to significant musculoskeletal risks, as assessed by the OHS.

Based on these criteria, two tertiary hospitals with specialized acute care, psychiatry, long-term and primary care were selected.

### Recruitment of participants

To recruit participants, informative sessions were performed at each cluster before disclosing whether they would be allocated to the intervention or control group, and informed consent and the baseline questionnaires were obtained from each participant worker. After these documents were filled in and returned, the units were randomized and informed about their condition of being intervention or control group, and the intervention started. Follow-up questionnaires are administered at six and 12 months. The questionnaires are anonymous to promote participation and worker’s confidence towards the intervention. Anonymized data related to sickness absence are collected for the period from 1 year before the intervention until the end of the study.

### Eligibility criteria

The inclusion criteria are nursing staff (nurses and aides), including employees on sickness absence, who voluntarily accept to participate. Workers with a temporal contract during a short period, working in several units and on sabbatical leave are excluded.

### Intervention

The intervention covers the three levels of prevention (primary, secondary and tertiary prevention), lasts for 1 year and is implemented to the control group thereafter.

For primary prevention of occupational risk factors, a standardized procedure of participatory ergonomics named ERGOPAR, developed and previously piloted in Spain is used [39]. A working group (ERGO group) is organized for each hospital unit, including a specialist in ergonomics, the unit supervisor/s, one referent worker for each shift (morning, afternoon and two night shifts) and one workers’ union representative. This ERGO group is responsible for the development and implementation of the intervention, and receive basic training in ergonomics and participatory methods. The intervention begins with the diagnostic phase, with the distribution of a previously validated self-completed questionnaire in which data on MSP and exposure to musculoskeletal risk factors at work are collected using specific risk assessment. This information is then analyzed and discussed by the ERGO group and a prioritized list of risk factors is developed. The next step is the treatment phase, in which the information collected in the questionnaires is shared and discussed with coworkers in the so-called prevention circles. These circles propose a prioritized list of preventive measures aimed to avoid or reduce the identified ergonomic risk factors. This list may include structural, technical, organizational, training and/or information measures for improvement, and workplace modifications. The OHS coordinates the implementation of these measures along with the corresponding department managers. Based on the previous available experience with the ERGOPAR method [22], the time required to complete all phases of the intervention in each work unit has been estimated from six to 12 months.

Secondary and tertiary prevention is carried out through a tailored case management intervention. Participant workers are voluntarily referred to case management either by themselves, by proposal of their supervisor or a physician of the OHS. Workers with a serious underlying organic pathology are excluded from this intervention component and managed according to standard medical practice. A trained case manager assigns participants to three strata of management and treatment, according to their level of risk for persistent musculoskeletal symptoms: low, medium or high. This profile is obtained by telephone interview using a questionnaire made of validated tools to generate a risk profile and that assesses the presence of radiated pain, comorbidity, limitations to carry out daily activities, discomfort derived from pain, fear of movement, beliefs and negative expectations regarding the prognosis of pain, the presence of anxiety and of other mood disorders [23, 24, 38, 40–43]. Workers assigned to the low-risk group attend an education session on health beliefs related to MSP. Workers assigned to the medium and high-risk groups receive specific and tailored treatment including rehabilitation, physiotherapy and cognitive-behavioral therapy. In parallel, cases may be discussed at the weekly clinical session with members of the OHS to evaluate possible specific needs at work, as job adjustments or improvements to help workers to stay and/or early return to work. The case manager contacts all workers regularly to carry out a motivational telephone follow-up, and also coordinates the services and plan the sessions.

An evidence-based program to promote healthy lifestyles at work is also part of the intervention. This program is addressed and offered to all nurses and aides of the participating intervention units, and application is voluntary and free. It includes: (1) mindfulness training, defined as a self-regulation approach to stress reduction and emotional management [44], consisting of an adapted course of 4 sessions of 2 h each on MBRS (Mindfulness-based Stress Reduction) training which has been shown to be effective in healthcare workers [29]; (2) Nordic Walking training, defined as a walking technique that uses specially designed poles to actively involve the upper body and arms with wide scientific evidence of its benefits on various health outcomes, including MSP and MSDs [30], offering a program of 12 sessions of 1.5 h/session during 12 weeks; and (3) healthy eating based on the Mediterranean diet, as one of the healthiest dietary models that currently exist [31, 32] consisting on a 3 h session and a web platform.

Finally, all components of the intervention are integrated and require coordination by a champion who acts as a leader and facilitator of the intervention, organising and leading the work to be developed by the study team. His/her tasks involve communication, meetings planning (i.e. informative sessions, and research team meetings), organization of the health promotion activities (i.e. calendar planning), collection and data processing and writing of reports.

### Occupational health care as usual

During the intervention period, the OHS of each participating hospital keeps providing the standard occupational health practices for both the intervention and control units. These practices include usual occupational risk assessments, investigation of occupational injuries, health surveillance, smoking cessation, training, information and expert assessment in occupational health at all levels in the hospital (i.e. managers, supervisors, workers), as well as the usual support program for return to work mainly focused on interventions related to workplace adaptations, clinical support and management of permanent disability.

### Measurements and procedure

Data are collected by standardized, validated questionnaires, processed in registers and a sample was double-checked to identify inconsistencies and errors. Self-reported questionnaires are administered at baseline, six and 12-month follow-up. Details on process evaluation are also stored in standardized registers, and double-checked. Data of sickness absence will be extracted from the company registries.

### Primary outcomes

#### Prevalence of musculoskeletal self-perceived pain

The Spanish adaptation of the Nordic Questionnaire from the ERGOPAR Method [45] is used to measure self-perceived MSP in the neck, shoulders and upper back, low back, elbows, hands, legs, knees, and feet, through the dichotomous question “Do you have discomfort or pain in this area?”. Data are collected at baseline, six and 12-month follow-up.

#### Sickness absence

Data on episodes and duration of sickness absence due to a musculoskeletal condition (MSP or MSDs) are collected from the company registries and the OHS during the study period and 1 year before the intervention.

### Secondary outcomes

#### Work functioning

Work functioning is measured at baseline, six and 12-month follow-up using the Work Role Functioning Questionnaire-Spanish Version (WRFQ-SpV) [46–48]. This tool is a self-administered questionnaire that measures perceived difficulties in performing one’s job due to health problems [49] and consists of 27 items divided into five subdomains: work scheduling demands, output demands, physical demands, mental demands, and social demands. The score of this questionnaire ranges from 0 to 100, being 100 the maximum score (having 100% of your functional capacity).

#### Organizational preventive culture

Organizational preventive culture is measured by the Institute for Work & Health Organizational Performance Metric (IWH-OPM) [50]. The IWH-OPM is an evidence-based, eight-item questionnaire used to help organizations assess and improve their health and safety performance and is measured at baseline, six and 12-month follow up.

### Process evaluation

The intervention process will be evaluated based on previous evidence and indicators for process evaluation [51, 52]: recruitment, context, reach, dose delivered, dose received, fidelity and satisfaction. Additionally, the stakeholder’s role will also be included (implementation strategy). Process evaluation data will be collected by means of questionnaires, the champion registries and qualitative approaches (discussion groups with researchers and participants). Recruitment, context and reach indicators will be available in the intervention and control group; dose delivered, dose received, fidelity and satisfaction will only be available for the intervention group since these indicators refer to various aspects of the intervention itself.

Context information will be collected through discussion groups and also, at six and 12-month follow-up with three questions related to the aspects that affect their usual workload (improvement in the manual mobilization of patients, technical aids, and load handling). Recruitment, reach, dose delivered and dose received, and fidelity (of the intervention and adherence of the participants) data will be extracted from the champion registers. Recruitment refers to the procedures used to approach and attract prospective program participants, as defined as the proportion of possible workers who agreed to participate in the study signing the informed consent at baseline. Reach can be defined as the proportion of the intended target audience that participates in an intervention, according to the intervention and control group reach and will be calculated considering the proportion of people who answered the questionnaires from those who signed the informed consent. For each component of the intervention, reach will be calculated as the proportion of people who have participated in each one. Dose delivered will be calculated as the number of hours of offered services, and dose received as the extent to which participants have actively participated in each component of the intervention. The intervention and participants fidelity will also be assessed. We operatized the fidelity of the intervention as a ratio between the planned and the developed one, and the fidelity of the participants as adherence through the proportion between their attendance and the dose delivered. Questions on satisfaction are self-administered at the end of each component of the intervention and at the 12-month follow-up questionnaire, through the questions “Have you met your expectations?” and “in general, what is your satisfaction?” on a scale of 1 to 10, being 10 the maximum satisfaction. Qualitative data from the discussion groups will be used to identify the key points and the possible improvements, as the implementation strategy indicator.

### Economic evaluation

A cost-utility and cost-effectiveness analysis will be performed from the societal and the National Health System perspectives. The cost-utility analysis will be conducted to analyze changes in quality-adjusted life years (QALYs) measured by EQ-5D-3 L [41], and the corresponding costs for each perspective. Moreover, the cost-effectiveness analysis will compare changes in MSP to the costs for each perspective. The national health system perspective will include the direct costs of the Spanish public health services (direct costs of the disease: visits to the GP, specialists, diagnostic tests and medication) and the costs of the intervention; the societal perspective will include all these costs and also the loss of production (indirect costs). Direct costs will be estimated based on administrative data from the clinical registers. Indirect costs will be estimated using the human capital approach from sick leave (company registries). Costs of the intervention (i.e. time of experts) will be obtained from the study register and will become unit costs according to the corresponding collective agreements.

### Randomization

Nursing staff (nurses and aides) of the hospital units with higher exposure to musculoskeletal risk due to ergonomic risk factors at the hospital unit and the type of patients (medium and high dependency) are selected. An independent researcher assigns the clusters to the intervention group or to the control group by simple randomization stratified by center.

### Blinding

In this cluster randomized trial blinding was not possible. The condition of being included in the intervention or the control group cannot be blinded but the clusters are randomized after signing informed consent and completing the baseline questionnaire. The services provided and the participating OHS professionals cannot be blinded because they are involved in the implementation of the intervention.

### Data analysis

Statistical analyses adapted for cluster-randomized controlled trials will be conducted. Descriptive analyses of the participants’ characteristics and comparisons between the intervention and control clusters will be performed. The generalized estimation equations (GEE) procedure will be used for the analysis of the MSP, work functioning and organizational preventive culture comparing the difference from the baseline to the 12-month follow-up of the intervention group, with the difference from the baseline to the 12-month follow-up of the control group. The models will be adjusted by the cluster design and by the potential confounders. The incidence of sickness absence will be analyzed through a logistic regression model, taking as a reference the episodes started the year before the intervention and the control group. A Cox proportional hazard model will estimate the hazard ratio of returning to work earlier after the intervention in the intervention group compared to the control group.

Statistical analyses will be performed with STATA 13 (StataCorp, 2013. Stata Statistical Software: Release 13. College Station, TX: StataCorp LP).

### Statistical power

The sample size estimation is based on the prevalence MSP estimated at 80% for healthcare workers [3], the impact of the intervention expecting a reduction of a 20% in the prevalence of musculoskeletal pain [53, 54], alpha values (type I error) = 0.05, statistical power = 0.80 and interclass correlation coefficient (ICC) = 0.05. Applying these criteria, we obtained a minimum sample of 164 subjects, 82 in each group. The units of the participating hospitals have a varied number of workers (20 to 60). Taking into account the sample size calculations, 8 units have been randomized.

## Discussion

A multifaceted intervention on MSP in hospital nursing and aide staff has been designed and described. The impact on MSP in the population shows that this is one of the main health challenges that must be addressed in Spain and similar countries in terms of health, work and economy [2, 3, 6].

### Methodological considerations

The randomized controlled trial (RCT) is the basic methodological paradigm for the evaluation of health interventions. Randomization guarantees that the assignment of a work unit to the intervention or control group is exclusively due to chance, thus avoiding effects of confounding and selection biases. The availability of a control group makes it possible to distinguish between epidemiological and/or statistical associations and cause-effect relations.

The INTEVAL_Spain has some strengths that make it unique in occupational health. First, it is a multifaceted intervention that includes the three preventive levels (primary, secondary, tertiary) simultaneously to reduce MSP in workers. Secondly, it has a multidisciplinary, de-medicalized and participatory approach, as it requires the involvement of the main agents in the company (managers, workers, technicians). And third, this study places special emphasis on the evaluation of results, process, and economics, and uses quantitative and qualitative methods for collecting data to achieve a comprehensive and accurate assessment at all levels of the intervention. In addition, this study places special emphasis on the evaluation of results, process and economics, and consists of mixed methods of collecting data (qualitative and quantitative) to achieve a comprehensive and accurate assessment at all levels of the intervention.

Our study has also potential limitations that we need to consider. There may be a participation bias, since workers with MSP could be more interested in participating, and otherwise, workers with good health could not feel the need of participating. Therefore, a sensitivity analysis comparing the sociodemographic variables of the participants and non-participants of each cluster will be carried out in order to quantify it. Moreover, there could be contamination, which could result in an underestimation of the effectiveness. However, clusters are different units located in different buildings and/or different floors, and therefore may not have much contact between them. Also, as it is a specific population (nurses and aides of public hospitals), the external validity of the results concerning the working population may be limited. Finally, questionnaires are anonymous and an identifier is not available. The research team made this decision to encourage participation and make sure that participants were feeling comfortable with the study, since the questionnaires were self-completed in the workplace and included some personal questions, as well as questions about the relationship with the supervisors. However, we are aware that this decision could limit the statistical power and entail an underestimation of the results.

### Possible impact of results

The preventive intervention evaluated in the INTEVAL_Spain project is characterized by its flexibility, efficiency and capacity to adapt to different companies’ needs. In addition, it is designed to optimize and make most of existing prevention resources in companies cost-effective. The whole project, in fact, is based on the sum of capacities of the different participants: managers, workers and their representatives, researchers, and occupational health specialists, taking advantage of the strengths and potentials of each of these agents and is carried out in close collaboration between the participating companies. This condition is both an opportunity and a challenge. An opportunity, since the direct interaction with the company’s agents in the development of the research strengthens the relationships among research centers, researchers themselves and the company, and facilitates the transfer of results to their direct users and targets. It is also a common challenge in occupational health intervention studies, as it is necessary to achieve the adjustment between the methodological requirements of the research and times, expectations and needs of the productive activity in the company.

This study aims to promote a change of orientation and a new paradigm for the prevention and management of MSP and associated sickness absence. The idea of implementing these combined and non-medicalized interventions in the OHS in a sustainable way over time could facilitate access for an early management of MSP at work, improve their health and be cost-effective.

## Abbreviations

MSDs: Musculoskeletal disorders
MSP: Musculoskeletal pain
OHS: Occupational Health Service
